# A Pilot Nurse-Led Tele-Counseling Intervention to Parents of Children With Cerebral Visual Impairment on Adherence to Eye Activities During COVID-19 Pandemic: A Pre-experimental Study

**DOI:** 10.3389/fmed.2021.740265

**Published:** 2022-02-17

**Authors:** Sonam Dhiman, Sushma Kumari Saini, Shweta Chaurasia, Mona Duggal, Vaibhav Miglani, Srishti Raj

**Affiliations:** ^1^National Institute of Nursing Education, Post Graduate Institute of Medical Education and Research, Chandigarh, India; ^2^Department of Ophthalmology, Advanced Eye Centre, Post Graduate Institute of Medical Education and Research, Chandigarh, India; ^3^Department of Hematology, Post Graduate Institute of Medical Education and Research, Chandigarh, India

**Keywords:** nurse-led counseling, tele-counseling, cerebral visual impairment, adherence, visual therapy, eye activities, COVID-19

## Abstract

**Aim:**

To assess the impact of a culturally appropriate and theoretically driven nurse-led tele-counseling intervention to parents of children with cerebral visual impairment (CVI) on the adherence to visual therapy advised by the ophthalmologists.

**Design:**

A pre-experimental design was used.

**Methods:**

Parents of children with CVI aged 2–9 years were enroled telephonically during the coronavirus disease 2019 (COVID-19) pandemic through the Pediatric Ophthalmology Clinic of a tertiary eye care center. Pre-assessment of participants was done telephonically as per the interview schedule. As per the protocol, the trained nurse-delivered tele-counseling intervention two times weekly for the first 2 weeks and weekly for the next 2 weeks *via* audio and video two-way tele-interaction with parents. A CVI information booklet was shared with parents *via* WhatsApp interface and individualized visual therapy was reinforced in accordance with the advice of the treating ophthalmologist. It was based on a thorough review of clinical records of the child, such as a detailed CVI questionnaire, history, and findings of clinical examination and neuro-visual behavior of a child. In addition, parents were encouraged to maintain a diary of the daily sessions of visual therapy and share recorded videos of their training exercises which were then evaluated and discussed with the ophthalmologist for any deviation. Outcome measures included adherence to eye activities for 7–8 sessions per day. Data were analyzed by using SPSS statistics for windows, version 20.

**Results:**

Overall, improvement of adherence to visual therapy was observed among children with CVI (*p* < 0.0001). Nearly half (47.6%) of participants adhered to 3–4 sessions/day and only 28.6% were adherent to the recommended 7–8 sessions/day.

**Conclusion:**

The pilot study demonstrated the potential of the nurse-led tele-counseling intervention to supplement the ongoing health treatment of patients in improving the adherence to eye activities among parents of children with CVI.

## Introduction

In children <3 years of age, cerebral visual impairment (CVI) is a major cause of bilateral childhood visual impairment (1). There has been a significant increase in the incidence of CVI from 36 per 1,00,000 in the late 1980s to 161 per 1,00,000 in 2003 (2, 3). This can be attributed to the higher survival rates in children with perinatal brain injury, neonatal infections, metabolic disorder, premature birth leading to hypoxic ischemic encephalopathy (HIE), periventricular leukomalacia (PVL), and many more (1, 4) may mean that more children are at potential risk for CVI.

There are no evidence-based guidelines to diagnose CVI. Diagnosis is based on visual assessment by an experienced pediatric ophthalmologist and the neuro-visual behavior assessment of the child combined with a thorough review of clinical records, such as CVI questionnaire, history, MRI findings, fundus examination, and findings of clinical examination. No standard therapy is in practice for pediatric CVI. Advanced care of preterm children, children with hypoxic-ischemic encephalopathy, and various methods of visual stimulation have been advocated as a treatment for CVI which may in the future reduce the incidence of this disorder and thus, may help in averting significant developmental impairment (5).

In a developing country, such as India, there is a lack of trained ophthalmologists, i.e., 1 ophthalmologist for every 90,000 people (6). This leads to a high patient load in the ophthalmology outpatient departments (OPDs). It is impractical to expect proper counseling of each parent of a child with CVI as they need long-term monitoring and adherence to visual therapy. Individualized visual therapy for each child is prescribed by an ophthalmologist but monitoring of adherence to prescribed eye activities performed by parents for their children with CVI on a regular basis is an extremely challenging task in OPDs. During the ongoing coronavirus disease 2019 (COVID-19) pandemic, the inability to assess the treatment compliance among parents of children with CVI became more pronounced. Moreover, queries of parents regarding the technique of performing eye activities remained unresolved.

Eye activities for visual therapy are complex and teaching them correctly to parents is important. A child with CVI has distinct preferences for bright colors (7) and tends to prefer looking at familiar objects as observed clinically in our healthcare center. Children may be able to use their peripheral vision more effectively than central vision, thus may face problems with visual motor coordination, calculating, reading, writing, and social interaction (8). Thus, a trained professional is needed who can understand the visual needs and teach the same to the parents of children with CVI.

Adherence to eye activities and follow-up for children with CVI form the backbone of management. Observations at our healthcare center have shown slow improvement in the visual behavior even after a long duration of treatment adherence thus parents lack the enthusiasm to comply with the treatment for long-time duration which as a result further slows the progression of visual function development of the child. An approachable and dedicated person, i.e., a nurse will be crucial during the long duration of visual therapy to solve the queries of parents in a timely manner and ensure adherence to eye activities.

Therefore, it is recommended that nurses, who are skilled to care for and counsel patients in all healthcare settings, can be professionally trained to teach, counsel, monitor, and reinforce the eye activities as advised by the treating ophthalmologist, thus serving as an important link between physicians and patients. Nurses can motivate, guide, and ensure adherence among the parents of children with CVI to perform eye activities by incorporating time in teaching parents more about the visual behavior of their child, queries, and their role throughout the visual therapy treatment.

Hence, a tele-counseling initiative for parents of children with CVI by a trained nurse was started as a service to bridge the gap of treatment compliance and ensure the timely resolution of queries of each parent during the COVID-19 pandemic. This is the first study as per our knowledge for assessing the impact of the nurse-led tele-counseling intervention on the adherence to eye activities in children with CVI.

## Materials and Methods

A prospective pre-experimental study was carried out at Pediatric Ophthalmology Clinic of a tertiary eye care center. The study conforms to the ethical norms and standards in the Declaration of Helsinki. The study was approved by the Institutional Ethics Committee and the trial was registered at CTRI with no. CTRI/2020/05/025063, dated: 8/5/2020. Informed consent was obtained through the WhatsApp interface from each participant. Total 21 children with CVI registered in Pediatric Ophthalmology Clinic from 2018 onward were enroled by using a total enumeration sampling technique.

### Training of Nurse Researcher

The nurse researcher, the study lead, was trained by a pediatric ophthalmologist to develop an understanding of CVI, causes, characteristics, types of neuro-visual behavior in children with CVI. A researcher was primed to fill up the CVI questionnaire (9). Based on clinical records and prior visual therapy recommended by an ophthalmologist, different eye activities delivered by a researcher for parents training were profiled after discussing with the ophthalmologist.

### Tools and Protocols

Tools and protocols were developed and validated by experts in the field of ophthalmology and nursing. The tools were pilot tested with five parents of children with CVI and found feasible and practicable. Pre-assessment was done by interviewing participants as per an interview schedule.

The two-way telephonic interaction included:

(1) An interview schedule consisting of: (a) the socio-demographic profile of child and parents and (b) the record of treatment adherence: the treatment adherence of last 1 month including the number of sessions and time spent per session in a day was assessed to determine the pre-intervention adherence maintained by parents. (c) A CVI questionnaire.
(2) Hospital clinical records included the clinical history of ophthalmic examination, filled CVI questionnaire (Annexure 3 in Supplementary Material), and neuro-visual behavior of the child.
(3) Daily diary to record the adherence to eye activities over a period of 1 month (Annexure 1 in Supplementary Material).

All this information was then reviewed for each child to understand the visual behavior and already prescribed eye activities.

The following operational definitions of specific terms used are described as follows:

- Nurse-led tele-counseling: It is the act of counseling and motivating the parents of children with CVI telephonically to perform regular eye activities for 7–8 sessions in a day with each session of 15–20 min, to facilitate vision and visual fixation through a culturally appropriate and theoretically driven counseling protocol.
- Adherence: It is the act to uptake eye activities by parents of children with CVI for 7–8 number of sessions per day and each session of 15–20 min. After 4 weeks of nurse-led counseling intervention adherence will be considered as: ≤ 2 sessions/day = poor adherence, 3–4 sessions/day = satisfactory adherence, 5–6 session/day = good adherence, and 7–8 session/day = excellent adherence.
- Eye activities: Eye-related activities to improve the deficit in neuro-visual behavior of a child with CVI. This includes eye activities related to the visual attention, visual fixation, latency, hand-eye co-ordination, etc. (Annexure 2 in Supplementary Material).

### Intervention

As per protocol, the trained research nurse delivered the intervention based on the specific visual needs of each child with CVI. The four steps included: (a) socio-demographic data, pre-assessment of treatment adherence of previous 1 month along with the queries and the technique of performing eye activities, and filling up of CVI questionnaire (9). (b) Assessment of the clinical records. (c) Profiling of eye activities for training of parents of children with CVI. (d) Counseling regarding eye activities to be performed specifically for parents of children with CVI.

Recorded videos were taken to assess the adequacy of performing already prescribed eye activities. Eye activities were profiled after a discussion with the treating ophthalmologist.

Eye activities related to domains of visual attention, visual fixation, latency, hand-eye co-ordination, spatial orientation, etc., were self-formulated by an ophthalmologist and were compiled in a CVI information booklet with a daily diary record sheet as a pdf version. Protocol for the nurse-led counseling intervention was developed by the research nurse under the collaborative guidance of the pediatric ophthalmologist, senior nursing, and public health faculty.

A nurse-led counseling was a two-way tele-counseling interactive session regarding CVI, causes, importance of eye activities, and teaching the child-specific eye activities to parents. For ready reference, the CVI information booklet in pdf version was given to parents. Accurate learning of parents was confirmed by evaluating and discussing the videos sent after the first telephonic interaction to the researcher by parents with the ophthalmologist. Any deviation in performing eye activities was corrected telephonically after discussing with the ophthalmologist and parents were motivated to maintain the diary of sessions of eye activities.

Any queries of parents regarding the technique of performing eye activities were further resolved in the follow-up calls. The follow-up calls were done over a period of 1 month, i.e., two times weekly for the first 2 weeks and weekly for the next 2 weeks. At the end of each follow-up call, parents were asked to share pictures of their daily diary over WhatsApp to ensure adherence and queries were resolved telephonically. Parents were free to call whenever needed and were motivated to adhere to the recommended 7–8 sessions/day of eye activities for 15–20 min/session during a 1-month time period.

**Figure 1:**
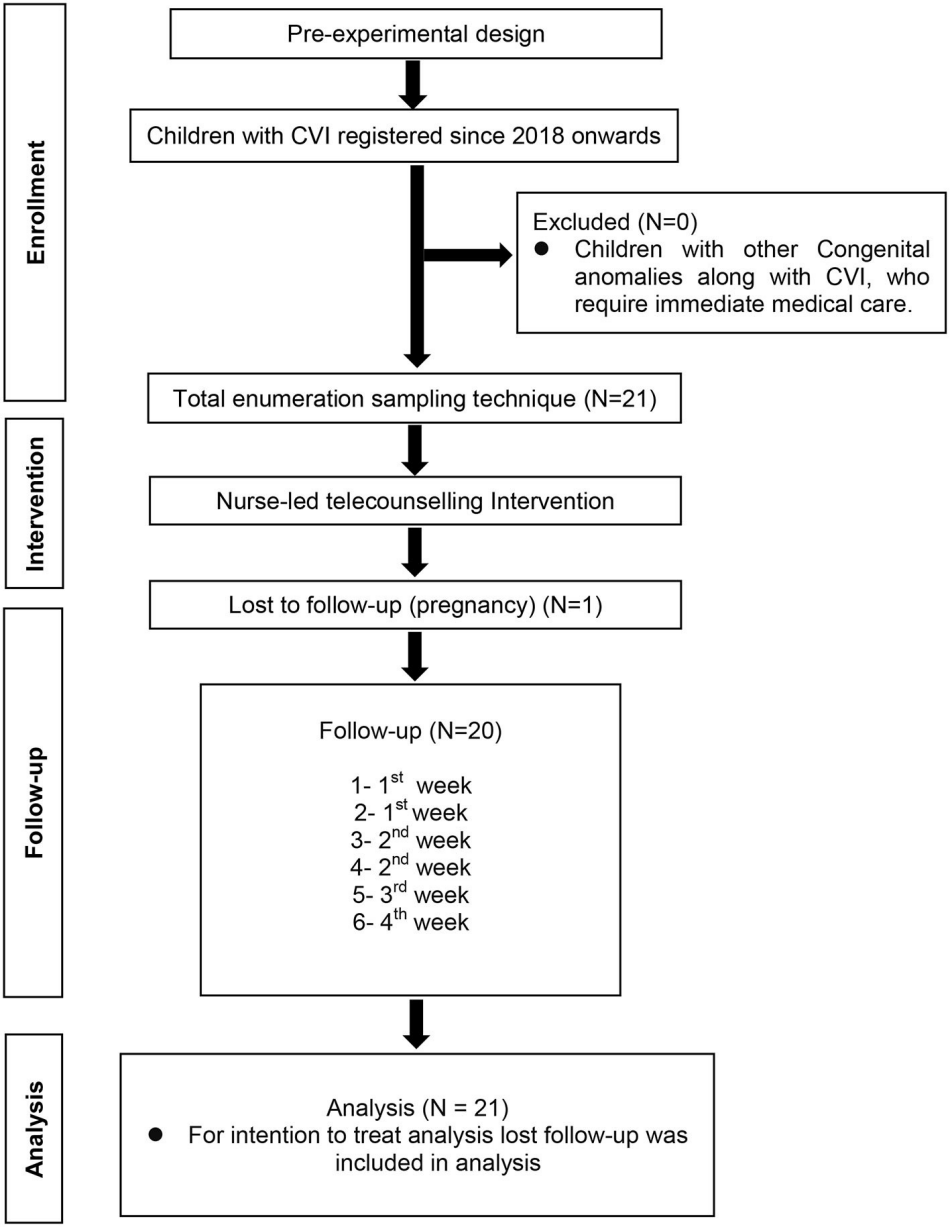
Consort diagram.

Post-assessment was done after 1 month of nurse-led tele-counseling intervention Adherence was assessed by the total number of sessions of eye activities done per day with the help of clicked photos of daily diary records sent after each follow-up and at the end of 1 month. After 1 month of nurse-led counseling intervention, ≤ 2 sessions/day was considered as poor adherence, 3–4 sessions/day as satisfactory adherence, 5–6 sessions/day as good adherence, and 7–8 sessions/day as excellent adherence. The categories were self-determined for the purpose of analysis. For the intention to treat analysis, a participant lost to follow-up was included in the final analysis (Figure 1).

## Results

Table 1 shows that the mean age of children enroled in the study was 3.03 ± 0.36 years and the majority (81%) were boys. Mean per capita income of the family was 32,021 ± 23,857 $ and nearly half were from social class I as per BG prasad scale (10). The details are elaborated in Table 1.

**Table 1:** Socio-demographic profile of children (*N* = 21).

**Variables**	***f*** **(%)**
**Age (in years)**	
1–2	10 (47.6)
2–3	8 (38.1)
≥4	3 (14.3)
**Gender**	
Male	17 (81.0)
Female	4 (19.0)
**Education**	
Not attending any school	20 (95.2)
Normal school	1 (4.8)
**Social class as per BG Prasad scale (per capita income)**	
Social class I (7,533 and above)	9 (42.9)
Social class II (3,766–7,532)	5 (23.8)
Social class III (2,260–3,765)	1 (4.8)
Social class IV (1,130–2,259)	3 (14.3)
Social class V (1,129 and below)	3 (14.3)
**Type of family**	
Nuclear family	16 (76.2)
Joint family	5 (23.8)
**Type of house**	
Pucca house	21 (100)
**Number of persons per room in house**	
One person per room	9 (43.0)
Two persons per room	10 (47.4)
Three persons per room	2 (9.6)
**Residence**	
Urban	8 (38.1)
Rural	13 (61.9)
Noisy	4 (19.0)
Calm	17 (81.0)

The age of mothers ranged from 21 to 48 years with a mean age of 30.10 ± 6.43 years. It was shown that 17 (81%) of the mothers were housewives. The age of fathers ranged from 25 to 50 years with the mean age of 33.75 ± 6.08 years and 7 (33.3%) fathers were in the occupation of skilled, craft, and machine operator as per International standard classification of occupations (Table 2).

**Table 2:** Socio-demographic profile of mother and father.

**Variables**	***f*** **(%)**	***f*** **(%)**
	**Mother**	**Father**
	***N*** **= 21**	***N*** **= 20**
**Age (in years)**		
21–30	14 (66.7)	6 (28.6)
31–45	6 (28.6)	12 (57.1)
≥46	1 (4.8)	2 (9.5)
Deceased	–	1 (4.8)
**Education**		
Illiterate	1 (4.8)	1 (4.8)
Primary education	1 (4.8)	–
High school education	4 (19.0)	2 (9.5)
Higher secondary education	3 (14.3)	5 (23.8)
Graduation and above	12 (57.2)	13 (61.9)
**Occupation**		
Managers/Professionals	2 (9.5)	6 (28.6)
Technicians and associate professionals	1 (4.8)	3 (14.3)
Service and sales workers	1 (4.8)	2 (9.5)
Skilled, Craft, and machine operators	–	7 (33.3)
Unemployed/Housewife	17 (81.0)	3 (14.3)

**Figure 2:**
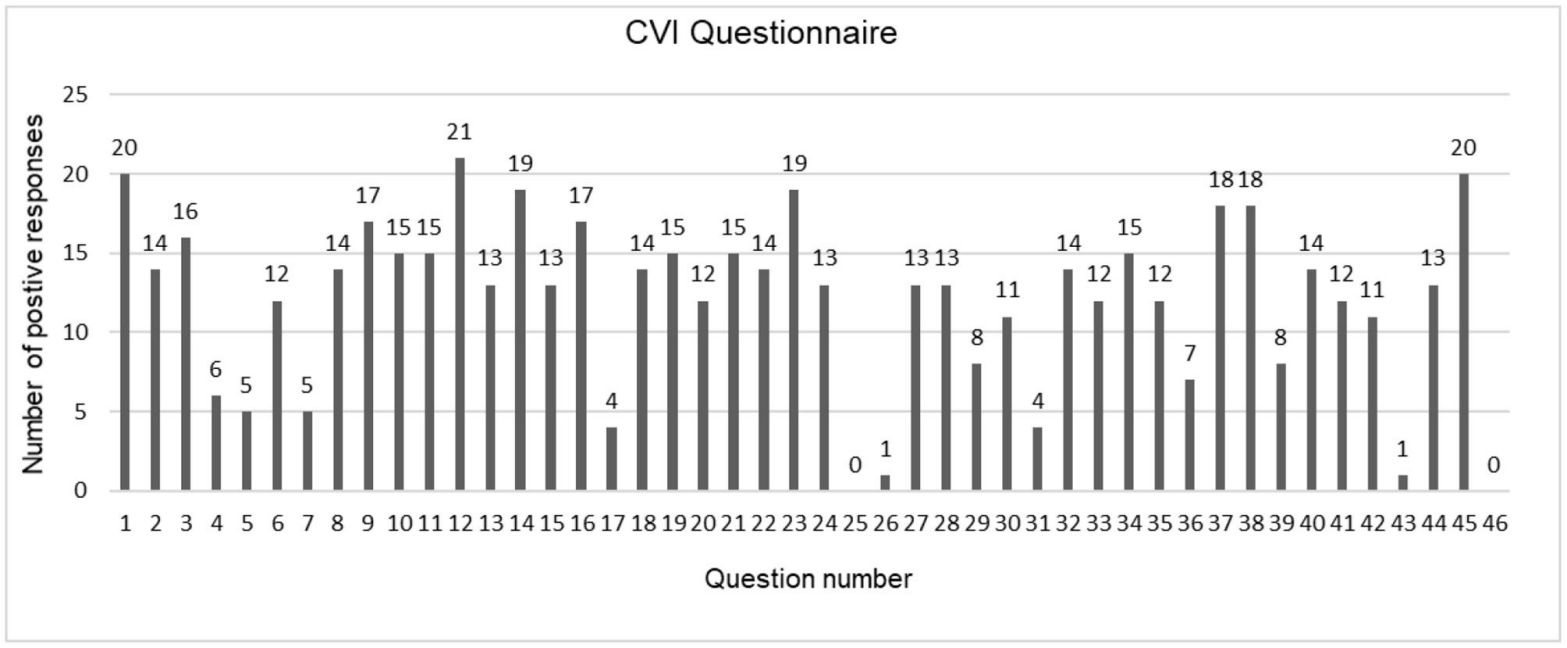
Visual needs among children with cerebral visual impairment (10).

The visual needs of children as per the CVI questionnaire (Annexure 3 in Supplementary Material) are depicted in Figure 2. While interviewing parents with questions from visual attitude domain of CVI questionnaire, it was noted that 10 (47.6%) children were not making eye contact, 14 (66.7%) children had a deficit in visual field and paid attention only to objects in the center of his visual field. The assessment of visual attention domain depicted that all 21 (100%) children needed more time to look at an object. Based on the results of CVI questionnaire (9), a better understanding of visual needs was attained and previous clinical records were used to teach the specific eye activity to the parents.

In pre-assessment, the treatment adherence as informed by the parents of a child in the previous 1 month, 17 (81%) parents were performing eye activities regularly and 4 were very irregular in performing eye activities. All of them had poor adherence to those who were performing. The mean time (in minutes) spent on the eye activity per day in the previous month was 21.67 ± 16.45 min/day (Table 3). The mean number of sessions of eye activities performed in the previous month before nurse-led tele-counseling ranged from 1 to 4 sessions/day with the mean of 1.76 ± 1.179 sessions/day.

**Table 3:** Treatment adherence of children.

**Variables**	***f*** **(%)**
Performed eye activities in last 1 month	17 (81.0)
Time spent on eye activity per day in previous month	
0 min	3 (14.3)
1–15 min	7 (33.3)
16–30 min	8 (38.1)
31–60 min	3 (14.3)
Number of sessions of eye activities performed in previous month	
≤ 2 sessions/day (poor adherence)	17 (81%)
3–4 sessions/day (satisfactory adherence)	3 (14.3%)
5–6 sessions/day (good adherence)	1 (4.8%)
7–8 sessions/day (excellent adherence)	–

**Figure 3:**
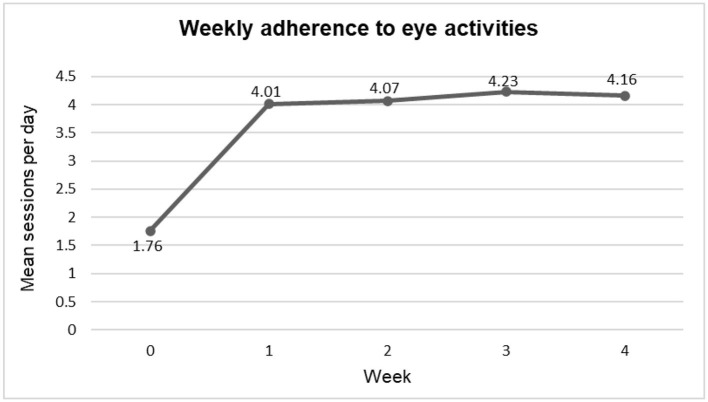
Weekly mean adherence to regular eye activities as per daily diary record.

The mean number of sessions performed by parents of children with CVI post-intervention is elaborated in Figure 3. As per the daily diary record, the maximum adherence was achieved after third week of intervention with a mean of 4.23 ± 1.913. One drop-out case at second follow-up was not performing eye activities (0 sessions/day) for the intention to treat analysis, it was taken in the final analysis.

A significant improvement in the adherence to eye activities (as per daily diary record) after 1 month of nurse-led counseling intervention as compared with baseline (0 week) was observed (*p* < 0.001, paired *t*-test) (Table 4).

**Table 4:** Comparison of mean score of baseline adherence with adherence after 1 month of intervention.

**Average number of sessions of eye activities done per day**	**Number of sessions**	***t*** **(***df***)** ***p***	**95% Confidence interval of the difference**	
	**Mean ±SD (Range)**		**Lower limit**	**Upper limit**
Baseline adherence (0 week)	1.76 ± 1.179	6.660 (20) <0.001	1.596	3.051
	(1–4)			
Adherence after 1 month	4.09 ± 1.782			
	(0–8)			

During pre-intervention, 17 (81%) parents were adhering to ≤ 2 sessions/day (poor adherence), 3 (14.3%) were adhering to 3–4 sessions/day (satisfactory adherence), and 1 (4.8%) was adhering to 5–6 sessions/day (good adherence). Post-nurse-led tele-counseling intervention, the adherence improved significantly. Post-intervention, 10 (47.6%) parents adhered to 3–4 sessions/day (satisfactory adherence) and 2 (9.5%) parents adhered to 7–8 sessions/day (excellent adherence). The results showed a significant improvement in the adherence to eye activities after 1 month of nurse-led counseling of intervention (*p* < 0.05, McNemar's test) (Table 5).

**Table 5:** Adherence to eye activities during pre-intervention and after 1 month of nurse-led counseling intervention.

**Sessions per day**	**Adherence to eye activities**		McNemar's test*
	**Pre-intervention**	**Post-intervention**	
	***f*** **(%)**	***f*** **(%)**	
≤ 2 sessions/day (poor adherence)	17 (81%)	3(14.3)	5.14 (1) 0.016
3–4 sessions/day (satisfactory adherence)	3 (14.3%)	10 (47.6)	
5–6 sessions/day (good adherence)	1 (4.8%)	6 (28.6)	
7–8 sessions/day (excellent adherence)	–	2 (9.5)	

* *For the purpose of applying McNemar's test poor adherence and satisfactory adherence were merged in one group. Good adherence and excellent adherence were merged into one group*.

A significant difference was observed (*p* < 0.005) in the mean value of the number of sessions of the eye activity done at weeks 1, 2, 3, and 4 (*post-hoc* repeated measure ANOVA) which indicated the adherence to eye activities improved after the nurse-led counseling intervention as compared with the baseline assessment. On comparing the adherence to eye activities at week 1 with subsequent weeks (2, 3, and 4 weeks), only minor improvement in adherence was observed in subsequent weeks which was statistically not significant. This indicated that parents sustained the adherence which they achieved at week 1 after nurse-led counseling intervention (Table 6).

**Table 6:** Comparisons of mean number of sessions of eye activities done during pre-intervention and at week 1, 2, 3, 4 after intervention.

**(I) average of week 1, 2, 3, 4**	**(J) average of week 1, 2, 3, 4**	**Mean difference (I–J)**	**Std. error**	Sig.**	**95% Confidence interval**	
					**Lower bound**	**Upper bound**
0	1	−2.252*	0.529	0.000	−3.77	−0.73
	2	−2.306*	0.523	0.000	−3.81	−0.80
	3	−2.469*	0.523	0.000	−3.97	−0.97
	4	−2.552*	0.529	0.000	−4.07	−1.03
1	2	−0.055	0.529	1.000	−1.57	1.47
	3	−0.218	0.529	1.000	−1.74	1.30
	4	−0.301	0.536	1.000	−1.84	1.24
2	3	−0.163	0.523	1.000	−1.66	1.34
	4	−0.246	0.529	1.000	−1.77	1.27
3	4	−0.083	0.529	1.000	−1.60	1.44

* *The mean difference is significant at the 0.05 level*.

** *Post-hoc repeated measure ANOVA*.

## Discussion

Visual therapy is recommended by an ophthalmologist for the improvement in neuro-visual behavior in children with CVI. Visual therapy requires regular adherence to eye activities for long durations, but this may be difficult to attain due to the short attention span of children with CVI and less understanding of parents regarding its importance. In an effort to attain adherence to visual therapy (regular eye activities), a dedicated professional is needed for motivating and resolving the queries of parents. Co-ordinated effort of neurologists, ophthalmologists, nurses, and parents in the form of a team has rewarding results for children with CVI and their parents. An interdisciplinary approach can be incorporated whereupon nurses can be given the task to counsel and resolve the queries of parents. Thus, enabling an ophthalmologist to incorporate time to treat more patients. This study presents a protocol for the nurse-led tele-counseling intervention pertaining to the adherence to eye activities for children with CVI during the COVID-19 pandemic. The nurse-led intervention is tailored to individual parent–child dyads, based on what eye activities they were performing based on the advice of the doctor and reinforcing the adherence to those eye activities. The combined results suggested that the nurse-led tele-counseling intervention during the pandemic era benefitted the children with CVI with timely access to healthcare and ensured adherence to the treatment.

At the baseline, all children and parents had received the minimum standard of treatment (eye activities) from an ophthalmologist to parents on the increased interaction with the child and performing eye activities for the improvement in visual behavior. Parents were given advice on the kind of toys they need to use during eye activities. On assessing the previous 1-month adherence practices of parents, the mean adherence was 1.76 ± 1.179 sessions/day against the recommended 7–8 sessions/day that indicated poor adherence to eye activities among parents of children with CVI.

An urgent need was felt to adopt the adherence strategies for the adherence to visual therapy in children with CVI for better management of limited time of the practitioners. Reasons for the non-adherence included the complexity of eye activities to be performed regularly for a longer duration with very little improvement in visual outcome in the child. Hence, the nurse-led tele-counseling intervention focused on regular motivation and follow-up by a professional nurse. A study in children with amblyopia showed that nurse-led intervention helped in adherence to the patching therapy (11). Thus, adherence to visual therapy could be well-attained by collaborative efforts by nurses and benefited in a similar way in children with CVI.

The adherence to eye activities for eye contact, latency, visual fixation, follow, and hand-eye coordination should benefit the children with CVI as seen in the treatment adherence studies (11). As per the visual needs of each child, eye activities to be delivered were profiled and intervention was delivered by a nurse after didactic training from an ophthalmologist. One-time learning may not be retained for longer duration, and the availability of the booklet in pdf version for ready reference helps in improving the adherence to eye activities. It has been seen in some studies that an educational cartoon story, a reward calendar, and an information leaflet for parents are beneficial in improving compliance with therapy in children (12). In our study, a CVI information booklet in pdf version with a daily diary record sheet was sent to parents through WhatsApp interface due to the prevailing COVID-19 pandemic.

Our study findings depicted that the mean number of sessions of eye activities performed per day improved significantly after 1 week of intervention and this achievement was sustained thereafter due to regular follow-up telephonic calls and motivation given by the nurse. Earlier, no telephonic follow-up initiatives were practiced by healthcare professionals. Moreover, irregularity in OPD follow-up was seen among parents of children with CVI as they were not reporting on the given date of follow-up. This led to the non-compliance of visual therapy and predisposed the child to delayed improvement in visual behavior. Regular motivation and clarification of queries by the nurse through regular telephonic follow-up calls led to the attainment of satisfactory adherence after 1 month of intervention from poor adherence as observed during pre-assessment. The telephonic follow-ups bound the parents by moral responsibility to comply with visual therapy (as verbalized by one parent). The results exhibited a favorable turnout of nurse-led tele-counseling intervention for children with CVI pertaining to treatment adherence.

Literature shows that documentation by maintaining a daily diary record and regular telephonic follow-up calls improves treatment adherence significantly (13). In our study, a daily diary record sheet was used to assess the adherence to eye activities by parents of children with CVI, which helped in achieving significant improvement in adherence after a period of 1 month. the nurse-led tele-counseling intervention was successful in motivating the parents and achieving adherence to eye activities in children with CVI. Incorporating it in regular practice through nurses in healthcare settings and in the community will have a fortunate turnout for children with CVI. “Nurse-led counseling clinic” can be run by the administration for counseling the parents to improve adherence to regular eye activities.

At baseline (0 week, pre-intervention), the number of sessions of eye activities performed per day ranged from 1 to 4 sessions/day with a mean of 1.76 ± 1.179 sessions/day and after 1 month of nurse-led tele-counseling intervention, it ranged from 0 to 8 sessions/day with a mean of 4.09 ± 1.782 sessions/day. A significant improvement in adherence to the recommended eye activities was observed after 1 month as compared with baseline (0 week) (*p* < 0.001, paired *t*-test). The results showed a positive influence of nurse-led tele-counseling intervention in sustaining the treatment adherence during the pandemic era when regular OPD follow-up was not possible in a tertiary healthcare setting.

## Limitations

The effect on visual outcomes cannot be commented on due to the short duration of the study as only 45 days were allotted for data collection in the Master's study period at our tertiary healthcare and the slow effect of regular eye activities. Due to recruitment challenges pertaining to the COVID-19 pandemic, the sample size was small as OPDs were limited to TeleOPD. A non-validated CVI questionnaire was used in this study due to the limited screening tools available for understanding the children with CVI. No new patients were registered in OPDs and a limited number of diagnosed children with CVI in our tertiary healthcare setting affected our statistical power.

## Conclusion

The nurse-led tele-counseling intervention demonstrated improvement in the adherence to eye activities among parents of children with CVI after a period of 1 month. The longitudinal trajectory findings showed the positive effect of the intervention. Regular motivation by a nurse through telephonic follow-ups calls can ensure compliance to the visual therapy in patients with non-emergency health conditions even during the pandemic era. First-ever nurse-led tele-counseling services can be incorporated in regular practice in healthcare settings to ensure accessibility of healthcare in a pandemic situation in the future. Thus, this nurse-led tele-counseling protocol can be used by nurses in their day-to-day practice to counsel the parents of children with CVI after attaining prior training in healthcare settings. This will bridge the gap between patient and healthcare, thus ensuring the treatment compliance as well as timely resolution of queries of each parent. The adoption of nurse-led tele-counseling in routine practice could bring additive benefits in improving the adherence to visual therapy and achieving favorable visual outcomes in children with CVI.

## Data Availability Statement

The original contributions presented in the study are included in the article/Supplementary Material, further inquiries can be directed to the corresponding author/s.

## Ethics Statement

The studies involving human participants were reviewed and approved by Institutional Ethics Committee, Post Graduate Institute of Medical Education and Research, Chandigarh. Written informed consent to participate in this study was provided by the participants' legal guardian/next of kin.

## Author Contributions

SD, SS, SC, and MD were involved in construction of tools and protocol of study. SD performed the study, gathered the data, provided intervention, and performed statistical analysis. SS, SC, MD, and SR were involved in revision of study protocol. SC involved in planning and supervised the work. SS and VM contributed to suggestions and corrections in the results. SS, SC, and MD contributed to final version of manuscript, revised, and suggested for corrections in the final manuscript. All authors contributed to the article and approved the submitted version.

## Conflict of Interest

The authors declare that the research was conducted in the absence of any commercial or financial relationships that could be construed as a potential conflict of interest.

## Publisher's Note

All claims expressed in this article are solely those of the authors and do not necessarily represent those of their affiliated organizations, or those of the publisher, the editors and the reviewers. Any product that may be evaluated in this article, or claim that may be made by its manufacturer, is not guaranteed or endorsed by the publisher.
