# Supplementary material for: A Pilot Nurse-Led Tele-Counseling Intervention to Parents of Children With Cerebral Visual Impairment on Adherence to Eye Activities During COVID-19 Pandemic: A Pre-experimental Study
Source: Front Med (Lausanne). 2022 Feb 17;8:740265. doi: 10.3389/fmed.2021.740265 (PMC8893197; doi:10.3389/fmed.2021.740265)
Supplement: Supplementary file 1 [file Presentation_1.PDF]

CVI Questionnaire (10)

1. VISUAL ATTITUDE

FIXATION

1. Absent eye contact
2. Cannot focus on persons nor objects
3. Tilts head to look at objects
4. Often stares at light sources (lights, open windows)

VISUAL FIELD

5. Falls frequently over clearly visible objects
6. Does not find his toy when he drops it
7. Bumps easily into something
8. Pays attention only to objects in the centre of his visual field

VISUAL ATTENTION

9. Cannot keep looking at objects or persons
10. Attention is fluctuating from moment to moment and from day to day
11. Abandons his play activity quickly
12. Needs more time than you'd expect to look at an object
13. Does not look spontaneously at an object, does not explore the room spontaneously
14. Needs encouragement to look at an object, explore the room
15. More toys perturb visual attention
16. Objects are looked at from a short distance
17. Sits right in front of the television

INFLUENCE FAMILIAR ENVIRONMENT

18. Scared or restless in unfamiliar environment (shop, street,..)
19. Does not find his/her parents when they stand further away
20. Clings to parents in an unfamiliar environment

2. VENTRAL STREAM

22. Does not recognize everyday objects such as an apple, bike, house, ball,…
23. Recognizes familiar objects only when they are drawn in color
24. Recognizes persons rather by listening to their voice, watching their posture than by
looking at their faces
25. Does not understand facial expressions (mad, sad, glad,..)
26. Does not find his way to the classroom, in his house (familiar environments)

3. DORSAL STREAM

27. Does not see level differences (stairs,..)
28. Cannot take the chocolate spread from the breakfast table without difficulty
29. Looks away when he takes the chocolate spread from the table
30. Has no interest for simple pictures
31. Has no interest for complex pictures
32. Looks only at details of a picture
33. Cannot find his teddy bear (or equal) amongst other cuddly animals
34. Does not find the chocolate spread on the table
35. Does not find/recognize familiar persons in a crowd
36. Cannot estimate distances

4. COMPLEX PROBLEMS

37. Clumsy in: cutting, building stacks, tying shoelaces, making puzzles
38. A moving object/person attracts more attention than a stationary one

5. OTHER SENSES

39. Reacts faster to sound than to visual stimuli
40. Manipulates an object rather than to look at it
41. Always puts objects, toys in his mouth

6. ASSOCIATED CHARACTERISTICS

42. Cannot play memory games
43. Stops activity when there is too much to look at (eg in a busy environment)
44. Is generally anxious
45. Does not do his best for tasks for which he needs to look carefully
46. I often wonder: does he not want to look at things or is he not able to?
47. He tries to compensate by talking a lot
