# Supplementary figures and images for: A Pilot Nurse-Led Tele-Counseling Intervention to Parents of Children With Cerebral Visual Impairment on Adherence to Eye Activities During COVID-19 Pandemic: A Pre-experimental Study
Source: Front Med (Lausanne). 2022 Feb 17;8:740265. doi: 10.3389/fmed.2021.740265 (PMC8893197; doi:10.3389/fmed.2021.740265)

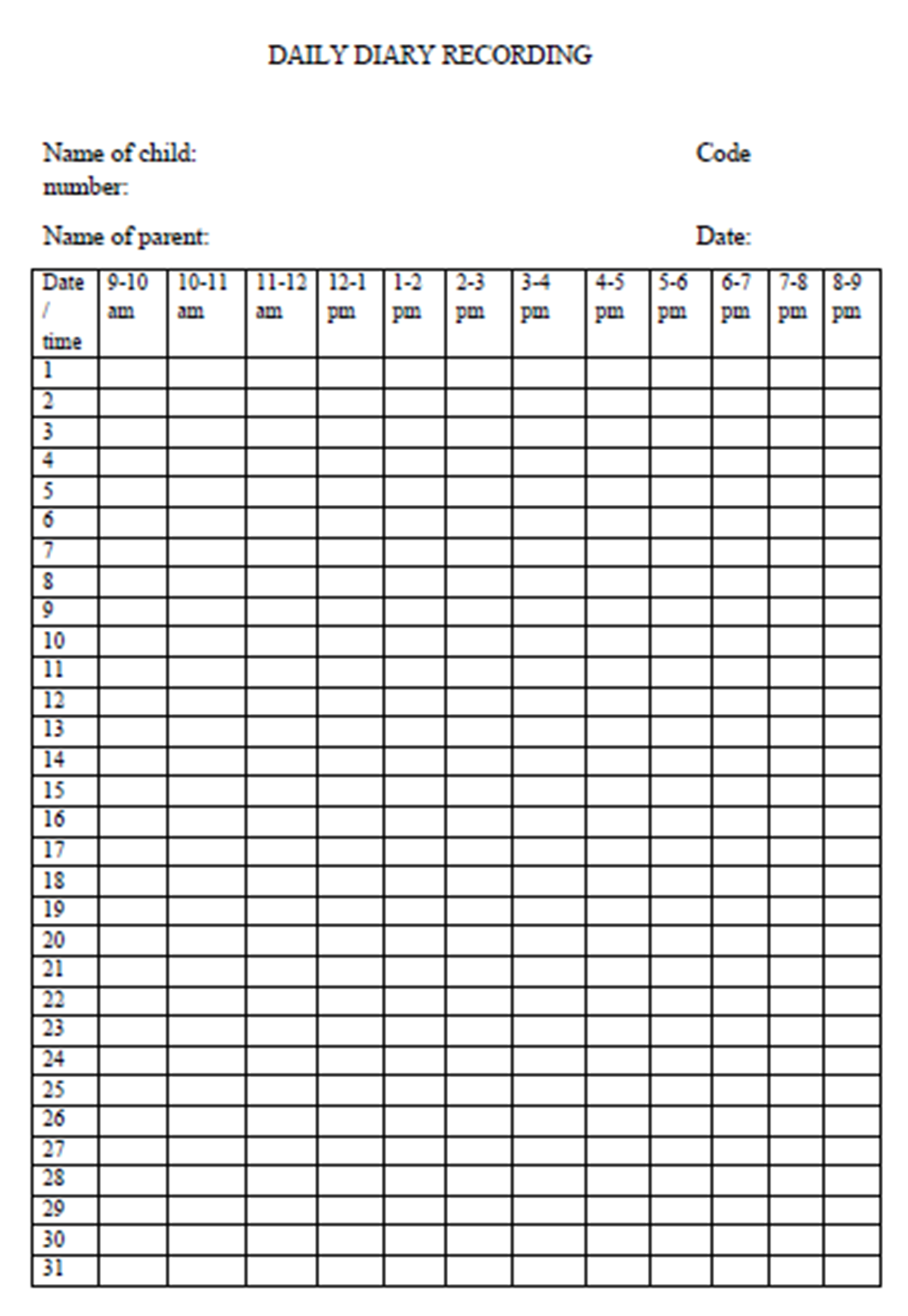

Supplement: Supplementary file 2 [file Image_1.PNG]

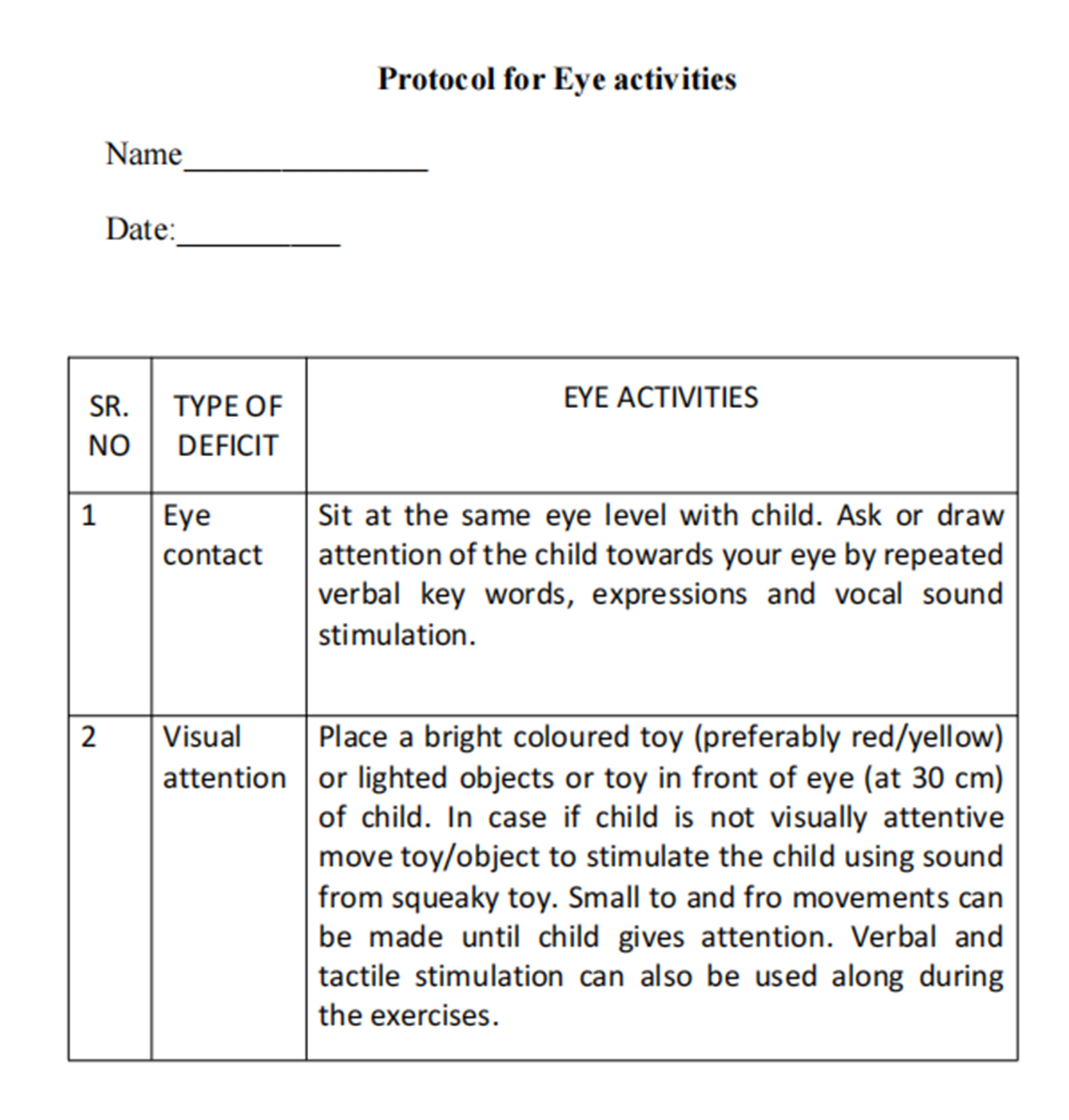

Supplement: Supplementary file 3 [file Image_2.PNG]
